# A genetic variant within *STS* previously associated with inattention in boys with attention deficit hyperactivity disorder is associated with enhanced cognition in healthy adult males

**DOI:** 10.1002/brb3.646

**Published:** 2017-02-09

**Authors:** Trevor Humby, Amelia Fisher, Christopher Allen, Meghann Reynolds, Annette Hartman, Ina Giegling, Dan Rujescu, William Davies

**Affiliations:** ^1^ School of PsychologyCardiff UniversityCardiffUK; ^2^ Division of Psychological Medicine and Clinical NeurosciencesMedical Research Council Centre for Neuropsychiatric Genetics and GenomicsSchool of MedicineCardiff UniversityCardiffUK; ^3^ Department of PsychiatryMartin Luther University of HalleHalleGermany; ^4^ Neuroscience and Mental Health Research InstituteCardiff UniversityCardiffUK

**Keywords:** 5‐choice serial reaction time task, coefficient of variation, intra‐individual reaction time variability, RRID: SCR_014794

## Abstract

**Introduction:**

The enzyme steroid sulfatase (STS) converts sulfated steroids to their non‐sulfated forms. Deficiency for this enzyme is associated with inattention but preserved response control. The polymorphism rs17268988 within the X‐linked *STS* gene is associated with inattentive, but not other, symptoms in boys with attention deficit hyperactivity disorder (ADHD).

**Methods:**

We initially tested whether rs17268988 genotype was associated with attention, response control, and underlying aspects of cognition, using questionnaires and neuropsychological tasks, in two independent cohorts of healthy adult males. In an additional analysis based upon existing data, the performance of mice with genetic or pharmacological manipulations of the STS axis under attentionally demanding conditions was investigated.

**Results:**

G‐allele carriers at rs17268988 exhibited reduced reaction time, enhanced attention, and reduced reaction time variability relative to C‐allele carriers. Mice with genetic or pharmacological manipulations of the STS axis were shown to have perturbed reaction time variability.

**Discussion:**

Our findings provide additional support for an association between rs17268988 genotype and attention, which may be partially mediated by reaction time variability; they also indicate that, in contrast to the situation in boys with ADHD, in healthy men, the G‐allele at rs17268988 is associated with enhanced cognition. As reaction time variability is a predictor of well‐being, rs17268988 genotype may represent a biomarker for long‐term health.

## 1 Introduction

The enzyme steroid sulfatase (STS), encoded by the X‐linked gene *STS*, cleaves sulfate groups from a variety of steroids (e.g., dehydroepiandrosterone sulfate) to convert them to precursors for a variety of estrogens and androgens that can elicit widespread and profound physiological effects (Mueller, Gilligan, Idkowiak, Arlt, & Foster, 2015). Studies in rodent models have implicated STS function in a number of aspects of cognition, including memory (Babalola et al., 2012; Johnson, Wu, Li, & Maher, 2000). Mice lacking the *Sts* gene, or given an inhibitor of the enzyme, display attentional deficits relative to wildtype or vehicle‐treated mice manifest as increased omission or commission errors respectively (Davies et al., 2009); interestingly, contrary to expectation, the former groups exhibit enhanced response inhibition relative to wildtype or vehicle‐treated mice (Davies et al., 2014). Somewhat consistent with these mouse data, males lacking a functional *STS* gene are at increased risk of developing attention deficit hyperactivity disorder (ADHD; particularly the inattentive presentation) but seem to exhibit normal levels of motor impulsivity (Chatterjee, Humby, & Davies, 2016; Kent et al., 2008). In the developing human brain, *STS* is highly expressed in brain regions important in attention and response control, notably the thalamus and the basal ganglia (Stergiakouli et al., 2011). Two independent genetic association studies examining the *STS* gene in boys from UK with ADHD identified the single nucleotide polymorphism (SNP) rs17268988 as being associated with inattentive symptoms, but not hyperactive or impulsive symptoms (Brookes et al., 2008; Stergiakouli et al., 2011); specifically, the G‐allele at this SNP was associated with a greater number of inattentive symptoms. No other SNPs around the *STS* gene showed evidence for association with disorder symptoms.

In the present study, we tested whether rs17268988 genotype was associated with aspects of attention or impulsivity in healthy adult males with a view to understanding how this polymorphism, or polymorphisms in linkage disequilibrium with it, may predispose to inattention in ADHD. Our main hypothesis was that possession of a G‐allele at this locus would be associated with impaired attention, but normal (or perhaps even enhanced) response inhibition. We subsequently tested whether our human findings were consistent with the previously obtained data from our mouse model studies.

## 2 Materials and Methods

### 2.1 Participants

Cohort 1 (*n* = 132 males aged 18–70 years (mean 37 ± 2 years), self‐reported as being cognitively healthy) was recruited via the Electronic Management System or Community Panel within the School of Psychology at Cardiff University, or from an internal University advert; recruitment and testing procedures were approved by Cardiff University School of Psychology Ethics Committee. Cohort 2 (*n* = 244 males, aged 18–70 years [mean 51 ± 1 years]) was recruited from around Munich, Germany and screened as described previously (Stergiakouli et al., 2011); recruitment, screening, and testing procedures were approved by the Institutional Review Board of the Ludwig‐Maximilians University of Munich. The two cohorts were assumed to be representative of the general populations of the UK and Germany respectively and were predominantly of White European ethnicity. Experiments were performed with the understanding and written consent of each subject.

### 2.2 Genotyping procedures

UK participants provided a saliva sample from which DNA was extracted using standard laboratory procedures; amplicons encompassing rs17268988 were produced by PCR (Forward primer: 5′‐CCAAAGGAGGGGTGTGTAAT‐3′; Reverse primer 5′‐GTAAAATCGCAAGCCCATGT‐3′) and sequenced. German participants were genotyped as described previously (Stergiakouli et al., 2011). As the *STS* gene is X‐linked, hemizygous males can only have either C‐ or G‐alleles at rs17268988.

### 2.3 Questionnaires

Cohort 1 completed an initial demographic questionnaire to take into account factors that could feasibly influence performance on the neuropsychological tests. Specifically, participants were asked to report age, handedness, and levels of tiredness (scale of 0–10, not tired to exhausted state respectively), stress levels (0–10, not stressed to extremely stressed respectively), recent caffeine and alcohol consumption, smoking status (i.e., nicotine consumption), and video‐game playing frequency. A subset of Cohort 1 (subset A, *n* = 65) were administered two questionnaires assaying attention and impulsivity: the 30‐item Barrett Impulsiveness Scale‐11 (BIS‐11; Patton, Stanford, & Barratt, 1995) and the 59‐item UPPS‐P Impulsive Behavior Scale (Lynam, Smith, Whiteside, & Cyders, 2006). BIS‐11 provided an overall measure of impulsiveness, together with sub‐scale measures of attentional, motor, and non‐planning impulsiveness. The UPPS‐P Impulsive Behavior Scale provided an overall measure of impulsiveness, together with sub‐scale measures of negative and positive urgency, lack of premeditation and lack of perseverance, and sensation‐seeking. The remainder of Cohort 1 (subset B, *n* = 67) were administered an 18‐item questionnaire based upon DSM‐IV criteria for ADHD, with level of agreement with each symptom being scored on a Likert scale from 1 (“never true of me”) to 5 (“always true of me”). This questionnaire provided an overall level of ADHD traits and relative levels of inattention and hyperactive‐impulsive traits (nine items each).

### 2.4 Neuropsychological tests

All Cohort 1 participants were administered two neuropsychological tests taxing attention, impulsivity and other relevant cognitive measures in the following sequence: (1) an adapted version of the Context‐Cuing Task (CCT; Verbruggen, Aron, Stevens, & Chambers, 2010) and (2) the freely available Psychology Experiment Building Language Test of Attentional Vigilance (TOAV) with default settings (Mueller & Piper, 2014; RRID:SCR_014794).

The cognitively demanding CCT was used to assess the ability to withhold a pre‐planned motor response (response inhibition) and the ability update a response‐set so as to execute an additional response. Participants were required to make speeded responses to a series of white arrow stimuli presented on a laptop screen (Toshiba Satellite Pro), pressing the “J” key for “<<<”and the “K” key for “>>>.” On a proportion of trials, the arrows would turn black after a variable period (the “signal”). The stimuli and signals appeared within two different task contexts: “stop” and “double.” During “stop” blocks, participants had to try and withhold their response upon presentation of the “stop signal.” Based on the horse‐race model (Verbruggen & Logan, 2009), a response will be successfully inhibited if completion of the stop process (triggered by the signal) occurs before completion of the go process (triggered by the stimulus). Increasing the delay between the stimulus and stop‐signal presentation (“stop‐signal delay,” SSD) reduces the probability of successfully stopping on stop‐signal trials (Verbruggen & Logan, 2009). The stop‐signal reaction time (SSRT) is a covertly obtained estimate of the latency of the stopping process (Verbruggen & Logan, 2009), and was calculated by an integration method (Verbruggen et al., 2010) utilizing an automated staircase tracking system in which the SSD is increased by 50 ms upon successful inhibition and decreased by 50 ms when inhibition is unsuccessful. During “double blocks,” participants had to execute a second response (space bar tap) when the “dual signal” appeared, immediately after their primary response to the white arrow stimulus. Participants respond more slowly to the dual‐signal when the time delay between the stimulus and dual‐signal, known as the “stimulus onset asynchrony” (SOA) is reduced, due to the existence of a “psychological refractory period” (PRP); the double blocks used fixed SOAs of 100, 250, and 400 ms. The PRP provides a measure of the delay in accessing response selection to the dual‐signal while individuals are completing central attentional processes for the initial stimulus (Pashler, 1984). The CCT task alternates pseudorandomly between stop and double blocks, permitting the reaction time and the proportion of mistakes made on the first trial following a switch (“RT Switch” and “Switch Cost” respectively) to be calculated. There exist four possible switches: stop‐to‐dual, dual‐to‐stop, stop‐to‐stop and dual‐to‐dual; a switch cost of 1 indicates that every response following a switch was incorrect, where as a switch cost of 0 signifies that no errors were made. Successful task switching requires the participant to update a response‐set, and to exhibit a degree of behavioral flexibility. One measure of a participant's reaction time variability, the “coefficient of variation” (CoV), was calculated by dividing the standard deviation of the reaction times by the mean reaction time (Jackson, Balota, Duchek, & Head, 2012); this metric has been used extensively in the literature and is easily calculated. Each participant completed one practice run followed by three complete runs, with each run lasting ~7 minutes and consisting of 12 “double” or “stop” blocks of nine trials each. The results from the three runs were averaged to calculate mean scores for each variable of interest.

In the TOAV, participants must respond to a black square that briefly appears within a white square. The stimulus is a target if it appears in the top portion of the white square; participants must respond by pressing the space bar. When the black square appears in the bottom portion of the white square it is a non‐target and participants must not respond. The test comprises of two halves: in the first half (Block 1) targets appear infrequently (infrequent condition, 72/320 trials), whereas in the second half (Block 2), targets are frequently presented (stimulating condition, 248/320 trials); Block 1 primarily taxes stimulus‐detection processes (attention) whereas Block 2 primarily taxes response‐inhibition processes (impulsivity). This test lasts approximately 24 min and as such taxes both sustained and selective attention. Main measures of interest included: omission errors (i.e., failure to respond to target presentation, reflecting attention), commission errors (i.e., response to a non‐target, reflecting attentional and impulsivity processes), correct reaction time (a measure of information processing and motor response time), CoV (standard deviation of reaction times divided by mean reaction time), and response sensitivity (*D*′), an indicator of the rate of deterioration in task performance, and of the accuracy with which targets can be discriminated from non‐targets (a measure of perceptual sensitivity).

Males from Cohort 2 underwent a battery of cognitive tasks (Winterer et al., 2010), of which the Continuous Performance Task (CPT) was most relevant to understanding attention. Key measures that were available from this sample included number of hits (i.e., responses to targets), false alarms (responses to non‐targets), and perceptual sensitivity (*D*′).

### 2.5 Animal studies

We re‐analyzed data from experiments previously reported in Davies et al. (2009). Briefly, wildtype (40,XY, *n* = 9) and *Sts*‐deficient (39,X^Y^*O, *n* = 11) male MF1 mice were tested on the 5‐choice serial reaction time task (5‐CSRTT) of attention, with light stimuli of 0.1, 0.3, 0.5 and 0.7 s presented pseudorandomly; wildtype male MF1 mice (*n* = 12) treated with both vehicle and the STS inhibitor Coumate (10 mg/kg, p.o.) in a randomized order were tested on 5‐CSRTT with light stimuli of 0.25, 0.5, 0.75 and 1.0 s presented pseudorandomly.

### 2.6 Statistical analysis

Data were analyzed using SPSS 20, and were tested for normality using Shapiro–Wilk test. Normally distributed data are presented as mean values ± standard error of the mean, and non‐normally distributed data as median values with 95% confidence intervals defined by bootstrapping. Human data were analyzed with two‐tailed *t* test (unless stated otherwise) if normally distributed (or if data could be normalized with natural log, reciprocal or square root transformation), or with Mann–Whitney *U* test if not normally distributed, with a between‐group factor of genotype (C‐ or G‐allele). Data comparing wildtype to *Sts*‐deficient mice were analyzed as above, while the effects of Vehicle or Coumate treatment on cognitive measures in the same mice were examined using paired t‐test or Wilcoxon Rank test for normal or non‐normally distributed data respectively. Categorical data were analyzed by chi‐squared test with Yates’ correction depending upon cell frequency. Correlations were performed using Pearson test (with normally distributed data) or Spearman test (with non‐normally distributed data). *p*‐Values <.05 were regarded as nominally significant.

## 3 Results

### 3.1 Genotyping

103/132 (78%) males in Cohort 1 possessed the C‐allele at rs17268988, and 29/132 (22%) the G‐allele. 175/244 (72%) males in Cohort 2 possessed the C‐allele, and 69/244 (28%) the G‐allele. These data are consistent with the previously obtained minor allele frequencies in boys with ADHD from UK and Ireland (21%–24%), and with data from HapMap CEU male samples from the general population (27%; Stergiakouli et al., 2011).

### 3.2 Demographics

C‐ and G‐allele carriers within the “discovery sample” (Cohort 1) were closely matched in terms of demographic variables that might feasibly have affected their performance on the neuropsychological tasks (Table 1). C‐ and G‐allele carriers within Cohort 2 were matched in terms of their age: C: 57 (95% CI: 53–59) versus G: 51 (95%: 48–58.5), *p* = .26.

**Table 1:** Demographic variables for healthy adult males recruited from UK with C‐ or G‐allele s at rs17268988

Demographic variable	C‐allele carriers (*n* = 103)	G‐allele carriers (*n* = 29)	Statistical comparison
Age (years)	28 (95% CI: 22.5–49)	23 (95% CI: 22–36)	*p* = .34
% right‐handed	89	100	*p* = .58
Tiredness level	4 (95% CI: 3–5)	4 (95% CI: 3–4)	*p* = .12
Stress level	3 (95% CI: 2–3)	2 (95% CI: 2–3)	*p* = .38
Caffeine consumption within past 4 hr	65	16	χ^2^(1) = 0.31, *p* = .58
Number of smokers	7	1	*p* = 1.0
Significant video‐game playing (>once per week)	38	11	χ^2^(1) = 0.01, *p* = .92

### 3.3 Questionnaire‐based measures

Levels of inattention and impulsivity within Cohort 1, as indexed by questionnaire‐based measures, were relatively low and comparable with previous data in healthy adult male populations (Cyders, 2013; Stanford et al., 2009). We found no evidence that males with C‐ or G‐alleles differed from one another in terms of their self‐reported impulsivity scores on the BIS‐11 or UPPS questionnaires (subset A, Table**** 2), or in terms of their self‐reported ADHD‐related traits (subset B, Table**** 3).

**Table 2:** Questionnaire‐based measures of impulsivity in healthy adult males recruited from UK with C‐ or G‐allele s at rs17268988 (Cohort 1, subset A). BIS‐11 (Barrett Impulsiveness Scale‐Version 11)

Impulsivity measure	C‐allele carriers (*n* = 47)	G‐allele carriers (*n* = 18)	Statistical comparison
BIS‐11			
Total score	65.0 ± 1.4	63.2 ± 2.8	*t*(63) = 0.63, *p* = .53
Attentional impulsiveness	17.7 ± 0.5	16.9 ± 1.1	*t*(63) = 0.70, *p* = .49
Motor impulsiveness	23.8 ± 0.5	22.6 ± 1.1	*t*(63) = 1.14, *p* = .26
Non‐planning impulsiveness	23.5 ± 0.7	23.7 ± 1.1	*t*(63) = −0.17, *p* = .87
UPPS‐P Impulsive Behavior Scale			
Total score	135.6 ± 2.7	132.1 ± 5.5	*t*(63) = 0.64, *p* = .52
Negative urgency	26.7 ± 0.8	25.2 ± 1.4	*t*(63) = 1.01, *p* = .32
Lack of premeditation	22.8 ± 0.8	22.2 ± 1.2	*t*(63) = 0.40, *p* = .69
Lack of perseverance	19.0 ± 0.6	19.8 ± 1.4	*t*(63) = −0.62, *p* = .54
Sensation‐seeking	41 (95% CI: 38.5–42.5)	41 (95% CI: 36–42)	*p* = .47
Positive urgency	27.6 ± 1.0	26.4 ± 2.0	*t*(63) = 0.62, *p* = .54

**Table 3:** Questionnaire‐based measures of ADHD‐related traits in healthy adult males recruited from UK with C‐ or G‐alleles at rs17268988 (Cohort 1, subset B)

ADHD‐related traits	C‐allele carriers (*n* = 56)	G‐allele carriers (*n* = 11)	Statistical comparison
Total score	39.3 ± 1.0	40.8 ± 1.5	*t*(65) = −0.63, *p* = .53
Inattention score	20.3 ± 0.6	20.8 ± 0.8	*t*(65) = −0.36, *p* = .72
Hyperactive‐impulsive score	18 (95% CI: 17–20)	20 (95% CI: 17–23)	*p* = .32

### 3.4 Context‐cuing task

The majority (89%) of participants from Cohort 1 understood the instructions for performing the CCT after a practice block, and exhibited behavioral performance in the task‐proper consistent with this. Interestingly, a higher proportion of C‐allele carriers than G‐allele carriers failed to learn the complex task (~12.5% vs. ~7%), perhaps consistent with enhanced general cognitive performance in the latter group. Across both types of Block (Dual and Stop), individuals possessing a G‐allele at rs17268988 exhibited a significantly shorter reaction time than individuals possessing a C‐allele at this locus; the former group also presented with shorter reaction times on the first trial of Stop Blocks after switching from Dual Blocks (Table**** 4). C‐ and G‐allele carriers performed equivalently on all other task measures.

**Table 4:** Context cuing task measures in healthy adult males recruited from UK with C‐ or G‐allele s at rs17268988

	C‐allele carriers (*n* = 90)	G‐allele carriers (*n* = 27)	Statistical comparison
Reaction time across Dual and Stop Blocks (ms)	470 (95% CI: 438–550)	428 (95% CI: 401–470)	*p* = .030
Coefficient of variation of reaction time across Dual and Stop Blocks	0.247 (95% CI: 0.236–0.265)	0.229 (95% CI: 0.200–0.253)	*t*(115) = 1.28, *p* = .203
Reaction time on Dual Blocks (ms)	455 (95% CI: 428–526)	415 (95% CI: 391–445)	*p* = .037
Coefficient of variation of reaction time on Dual Blocks	0.234 (95% CI: 0.224–0.249)	0.218 (95% CI: 0.196–0.235)	*t*(115) = 1.64, *p* = .104
Reaction time on Stop Blocks including erroneous responses on stop trials^a^, or excluding such responses^b^ (ms)	480 (95% CI: 451–590)^a^474 (95% CI: 446–564)^b^	430 (95% CI: 420–490)^a^436 (95% CI: 420–528)^b^	*p* = .046^a^ *p* = .038^b^
Coefficient of variation of reaction time on Stop Blocks including erroneous responses on Stop trials	0.243 (95% CI: 0.229–0.259)	0.232 (95% CI: 0.204–0.278)	*t*(115) = 0.82, *p* = .416
Stop signal reaction time (ms)	290 (95% CI: 276–309)	283 (95% CI: 255–349)	*p* = .426
Psychological refractory period (PRP; ms)	434 (95% CI: 362–524)	409 (95% CI: 268–571)	*t*(106) = 0.945, *p* = .347
Switch cost (stop‐dual)	0.905 (95% CI: 0.867–0.933)	0.933 (95% CI: 0.875–0.933)	*p* = .816
Switch cost (dual‐stop)	0.895 (95% CI: 0.867–0.909)	0.867 (95% CI: 0.800–0.900)	*p* = .089
Switch cost (dual‐dual)	1.000 (95% CI: 1.000–1.000)	1.000 (95% CI: 1.000–1.000)	*p* = .542
Switch cost (stop‐stop)	0.889 (95% CI: 0.834–0.889)	0.889 (95% CI: 0.833–1.000)	*p* = .753
Switch reaction time (Stop‐Dual; ms)	476 (95% CI: 456–531)	413 (95% CI: 403–495)	*p* = .095
Switch reaction time (Dual‐Stop; ms)	513 (95% CI: 457–561)	446 (95% CI: 403–493)	*p* = .024
Switch reaction time (Dual‐Dual; ms)	444 (95% CI: 432–505)	431 (95% CI: 417–462)	*p* = .112
Switch reaction time (Stop‐Stop; ms)	545 (95% CI: 490–587)	463 (95% CI: 416–532)	*p* = .056

Reaction time on Stop Blocks including erroneous errors on Stop trials.

Reaction time on Stop Blocks excluding erroneous responses on Stop trials.

### 3.5 Test of Attentional Vigilance

Test of Attentional Vigilance performance in the majority of Cohort 1 participants was successfully analyzed, although one G‐allele carrier did not complete the task due to fatigue. On Block 1, individuals possessing a G‐allele made significantly more correct responses than C‐allele carriers, made significantly fewer commission errors than C‐allele carriers, and had significantly lower variability in their reaction times than C‐allele carriers (Table 5); there was a significant positive correlation between the number of commission errors and the CoV across the individual genotypes (C: *r*_s_ = .538, *p* < .001 and G: *r*_s_ = .617, *p* < .001) and across the two genotypes combined (*r*_s_ = .592, *p* < .001). C‐ and G‐allele carriers did not differ on any other measures on Block 1. C‐ and G‐allele carriers did not differ significantly with respect to any measure in Block 2; the two groups did not differ significantly with respect to any measure across both Blocks 1 and 2 (Table 5).

**Table 5:** Test of Attentional Vigilance (TOAV) measures in healthy adult males recruited from UK with C‐ or G‐allele s at rs17268988

	C‐allele carriers (*n* = 103)	G‐allele carriers (*n* = 28)	Statistical comparison
Block 1			
Correct trials	316 (95% CI: 316–317)	318 (95% CI: 316–318)	*p* = .037
Commission errors	3 (95% CI: 2–4)	2 (95% CI: 1–2)	*p* = .036
Omission errors	0 (95% CI: 0–0)	0 (95% CI: 0–0)	*p* = .426
Correct reaction time (ms)	434.5 (95% CI: 412–446)	424 (95% CI: 401–462)	*t*(129) = 0.036, *p* = .972
Incorrect reaction time (ms)	434.5 (95% CI: 379–516)	430 (95% CI: 383–537.5)	.814
Coefficient of variation	0.195 (95% CI: 0.187–0.215	0.176 (95% CI: 0.150–0.203)	*t*(129) = −2.049, *p* = .042
*D*′	1.615 (95% CI: 0.352–1.858)	1.615 (95% CI: 0.000–1.858)	*p* = .410
Block 2			
Correct trials	307 (95% CI: 304–311)	306 (95% CI: 301–311)	*p* = .344
Commission errors	11 (95% CI: 9–14)	11 (95% CI: 8–16)	*p* = .886
Omission errors	1 (95% CI: 0–1)	1 (95% CI: 0–2)	*p* = .283
Correct reaction time (ms)	377 (95% CI: 354–391)	351 (95% CI: 328–377)	*t*(129) = −0.385, *p* = .701
Incorrect reaction time (ms)	323 (95% CI: 316–338)	301 (95% CI: 281.5–353)	*p* = .352
Coefficient of variation	0.201 (95% CI: 0.189–0.214)	0.204 (95% CI: 0.162–0.242)	*t*(129) = −0.293, *p* = .770
*D*′	1.858 (95% CI: 1.682–2.368)	1.742 (95% CI: 1.208–2.322)	*p* = .353
Combined Blocks 1 and 2			
Correct trials	624.5 (95% CI: 620–627)	624 (95% CI: 617.5–628)	*p* = .751
Commission errors	13 (95% CI: 12–16)	12 (95% CI: 8–20)	*p* = .633
Omission errors	1 (95% CI: 1–2)	1 (95% CI: 1–2.5)	*p* = .499
Correct reaction time (ms)	386.5 (95% CI: 370–411)	366 (95% CI: 348.5–394.5)	*p* = .411
Incorrect reaction time (ms)	346 (95% CI: 335–366)	327.5 (95% CI: 301–387.5)	*p* = .382
Coefficient of variation	0.224 (95% CI: 0.207–0.241)	0.211 (95% CI: 0.190–0.265)	*t*(129) = −0.586, *p* = .559
*D*′	1.034 (95% CI: 0.883–1.244)	0.895 (95% CI: 0.645–1.360)	*p* = .416

### 3.6 Continuous Performance Task

Based on the TOAV data for Cohort 1, we predicted that, in an independent sample, male carriers of a G‐allele at rs17268988 would exhibit enhanced cognitive performance relative to C‐allele carriers in an attentionally demanding CPT conceptually analogous to the TOAV. In Cohort 2, G‐allele carriers made more successful responses to targets (“hits”) and fewer erroneous responses to non‐targets (“false alarms”) relative to C carriers (79 [95% CI: 78–79] vs. 78 [95% CI: 78–79] and 1 [95% CI: 0–1] vs. 1 [95% CI: 1–1] respectively, one‐tailed *p* = .068 and *p* = .232). *D*′ for G‐allele carriers was significantly higher than that for C‐allele carriers (5.071 [95% CI: 4.807–5.268] vs. 4.932 [95% CI: 4.694–4.986], one‐tailed *p* = .0395) consistent with the improved stimulus detection sensitivity in the former group.

### 3.7 A re‐analysis of previously obtained mouse data

Given the data from the TOAV above, we re‐analyzed our previously published mouse data (Davies et al., 2009) to test whether the genetic or pharmacological manipulations had effects on reaction time variability under attentionally demanding conditions.

Relative to wildtype MF1 male mice, MF1 *Sts*‐deficient male mice exhibited evidence for reduced incorrect reaction time, reduced reaction time variability on incorrect trials, and reduced variability in reaction time across all responses (Table**** 6). When MF1 male mice were administered the STS inhibitor Coumate, they exhibited evidence for higher numbers of incorrect trials, and increased reaction time variability, relative to when they were administered vehicle (Table 7).

**Table 6:** Performance of wildtype (40,XY) and Sts‐deficient (39,X^Y^*O) MF1 mice on 5‐choice serial reaction time task under attentionally demanding conditions

	40,XY (*n* = 9)	39,X^Y^*O (*n* = 11)	Statistical comparison
Correct trials	29.8 ± 4.6	34.6 ± 4.0	*t*(16) = −1.268, *p* = .223
Correct reaction time (ms)	766.5 (95% CI: 639.6–864.1)	799.5 (95% CI: 717.3–917.5)	*t*(16) = 1.588, *p* = .132
Coefficient of variation on correct trials	0.390 (95% CI: 0.370–0.930)	0.435 (95% CI: 0.370–0.505)	*t*(16) = −1.039, *p* = .314
Incorrect trials	5.6 ± 1.1	6.2 ± 1.3	*t*(16) = −0.706, *p* = .490
Incorrect reaction time (ms)	2244 ± 247	1154 ± 101	*t*(16) = 5.802, *p* < .001
Coefficient of variation on incorrect trials	0.737 ± 0.058	0.423 ± 0.059	*t*(16) = 3.727, *p* = .002
All trials (correct and incorrect)	35.3 ± 4.3	40.8 ± 4.7	*t*(16) = −1.335, *p* = .201
Reaction time across all trials (ms)	1005.0 (95% CI: 858.5–1301.9)	842.0 (95% CI: 754.8–1014.9)	*t*(16) = −1.534, *p* = .145
Coefficient of variation on all trials	0.894 ± 0.089	0.491 ± 0.047	*t*(16) = 5.149, *p* < .001

**Table 7:** Performance of a group of MF1 male mice administered vehicle, or the STS inhibitor Coumate, on 5‐choice serial reaction time task under attentionally demanding conditions

	Vehicle (*n* = 12)	Coumate (*n* = 12)	Statistical comparison
Correct trials	36.9 ± 3.6	37.3 ± 4.4	*t*(11) = −0.096, *p* = .925
Correct reaction time (ms)	852.7 ± 74.0	837.9 ± 56.8	*t*(11) = 0.331, *p* = .746
Coefficient of variation on correct trials	0.635 ± 0.093	0.662 ± 0.079	*t*(11) = −0.269, *p* = .793
Incorrect trials	3.5 (95% CI: 1–9.5)	8.5 (95% CI: 7.0–14.5)	*p* = .032
Incorrect reaction time (ms)	1482.2 ± 178.3	1782.3 ± 143.6	*t*(9) = −1.408, *p* = .193
Coefficient of variation on incorrect trials	0.674 ± 0.068	0.774 ± 0.055	*t*(9) = −1.357, *p* = .208
All trials (correct and incorrect)	43.5 ± 4.9	48.8 ± 5.4	*t*(11) = −1.033, *p* = .324
Reaction time across all trials (ms)	943.9 ± 89.9	1084.9 ± 84.3	*t*(11) = −1.676, *p* = .122
Coefficient of variation on all trials	0.717 ± 0.070	0.867 ± 0.054	*t*(11) = −2.274, *p* = .044

## 4 Discussion

Mice and human males lacking a functional X‐linked *STS* gene exhibit attentional deficits, and the latter group are at increased risk of developing inattentive ADHD (Chatterjee et al., 2016; Davies et al., 2009; Kent et al., 2008). Previous evidence is available to show that the number of inattentive symptoms (but not impulsive or hyperactive symptoms) in boys with ADHD is associated with variation at the SNP rs17268988 within *STS* (Brookes et al., 2008; Stergiakouli et al., 2011). In the current study, we tested whether genotype at rs17268988 was associated with questionnaire‐based and neuropsychological measures of attention, response control and cognition in large samples of healthy males, and whether similar effects could be seen in mice with genetic or pharmacological manipulations of the STS system; these studies could potentially provide insights into the psychological and neural processes through which rs17268988 (or polymorphisms in linkage disequilbrium with it) could influence attentional (dys)function.

Converging data from two questionnaires assessing aspects of impulsivity (BIS‐11 and UPPS‐P) and a DSM‐IV ADHD criteria‐derived questionnaire assessing inattention and hyperactivity‐impulsivity indicated that rs17268988 genotype was not associated with large effects on self‐reported measures of inattention, hyperactivity, or impulsivity. The neuropsychological data however indicated subtle differences in cognition between individuals possessing C‐ and G‐alleles, notably on measures of reaction time, reaction time variability and response accuracy under attentionally demanding conditions; interestingly, the direction of these effects was counter to that which we hypothesized. Importantly, the between‐group differences were unlikely to be confounded by general factors (e.g., tiredness) that could influence task performance. rs17268988 genotype and cognitive performance may feasibly be causally related given previous animal model and clinical data explicitly demonstrating a role for STS in cognitive processes (including attention). It is also worth noting that we did not correct for multiple testing given that many of the measures assayed (both significant and not) are likely to be inter‐dependent; therefore, the aforementioned nominally significant findings, though somewhat replicable, should be treated with an appropriate degree of caution.

On the cognitively demanding CCT G‐allele carriers demonstrated significantly shorter reaction times than C‐allele carriers, possibly indicating superior information processing and/or more rapid motor responses. Consistent with the previous data in boys with ADHD, and with the questionnaire data, rs17268988 genotype was not associated with the main CCT measure of response inhibition (SSRT). Although G‐allele carriers tended to show reduced reaction times relative to C‐allele carriers on the TOAV, unlike in the CCT there was no significant effect of genotype; this discrepancy could potentially be explained by a ceiling effect in the less complex TOAV. We noted significant associations between cognitive measures on the most attentionally demanding component of the TOAV (Block 1) and rs17268988 genotype. Specifically, G‐allele carriers exhibited evidence for more accurate responding, and for responding in a more temporally consistent manner, relative to C‐allele carriers. As these two variables were significantly correlated with one another across genotypes, they may be causally related to each other, or alternatively, they may be affected by a common factor. C‐ and G‐allele carriers performed equivalently on Block 2 of the TOAV, an observation consistent with the notion that this allele is not associated with effects on impulsivity. Our finding of enhanced cognition in G‐allele carriers under attentionally demanding conditions was replicated in a large, independent healthy male sample; however, it should be appreciated that, given that the cognitive tests employed in Cohorts 1 and 2 differed, the extent to which the findings from Cohort 1 could be predicted to generalize to Cohort 2 (and hence, whether a one‐tailed *p*‐value is appropriate for Cohort 2 analysis) is arguable. We chose to use the freely available TOAV in order that replications of our study could be performed readily and cheaply, and we urge other researchers to test whether rs17268988 genotype shows a similar pattern of associations in alternative geographically and ethnically diverse populations.

The data presented above provides additional support for the idea that rs17268988 (or a linked polymorphism) is associated with aspects of attentional function but not response inhibition. However, there is a dissociation between the direction of effects seen in healthy individuals (poorer cognitive performance in C‐allele carriers), and in individuals with ADHD (greater inattention in G‐allele carriers). These contradictory findings could potentially be explained by: (1) an interaction between rs17268988 genotype, cognitive function and developmental stage (healthy males were aged 18‐70, boys with ADHD were aged 9–18 years); (2) an interaction between rs17268988 genotype, cognitive function and disorder‐specific factors (e.g., alternative genetic risk variants or environmental risk factors); (3) differences between clinical symptom scores obtained by child psychiatrists on the basis of parental reports and more objective neuropsychological measures; and (4) a combination of one or more of the above. With respect to the third possible explanation, there is existing evidence that behavioral inattention is not necessarily correlated with cognitive inattention (Jonsdottir, Bouma, Sergeant, & Scherder, 2006). A final, less likely, explanation for the dissociation is that the findings of enhanced cognition in healthy males carrying the G‐allele and/or the finding of impaired attention in boys with ADHD carrying the G‐allele are false positives arising from limited sample sizes or insufficiently stringent multiple testing correction.

Data from the TOAV suggested that, under attentionally demanding conditions, possession of a G‐allele at rs17268988 was associated with reduced variability in reaction time relative to possession of a C‐allele at this locus; the same pattern of effects was not seen when target frequency was high. We wanted to examine whether manipulations of the STS axis were associated with altered intra‐individual variability in reaction time (as indexed by CoV) under attentionally demanding conditions in mice. Through a re‐analysis of our previously obtained data, we found evidence consistent with the human data which suggested that both genetic and pharmacological manipulations of the STS axis influenced reaction time variability: loss of the *Sts* gene was associated with *reduced* reaction time variability in adult male mice (mainly mediated via greater consistency of responding on incorrect trials), while acute inhibition of the STS enzyme was associated with *increased* reaction time variability in adult male mice. The opposite direction of the genetic and pharmacological effects is intriguing, and could potentially be explained by the presence of compensatory processes in the gene deletion model which cannot occur in the case of acute enzyme inhibition. Differential behavioral effects in the genetic and pharmacological mouse models could also be explained by complete lack of the STS protein in the deletion model versus incomplete (~70%) inhibition of the enzyme in the pharmacological model (Nicolas et al., 2001), or by deletion of additional genes or genetic elements other than *Sts* in the genetic model (Trent et al., 2013; Trent, Fry, Ojarikre, & Davies, 2014) and possible off‐target effects in the pharmacological model (Ho et al., 2003). There is a growing body of evidence that pharmacological manipulation of the STS axis can influence the aspects of cognition and the underlying neural substrates (Yue et al., 2016).

Should the link between rs17268988 genotype and intra‐individual variability in reaction time be confirmed in follow‐up studies, this could have potentially important implications in terms of healthcare. Intra‐individual variability in reaction time is influenced by normal (Dykiert, Der, Starr, & Deary, 2012) and pathological aging (Phillips, Rogers, Haworth, Bayer, & Tales, 2013) and may be a predictor of early mortality (particularly through cardiovascular disease; Batterham, Bunce, Mackinnon, & Christensen, 2014); there have been suggestions that reaction time variability may represent a psychological marker of bodily system integrity (Ramchurn, de Fockert, Mason, Darling, & Bunce, 2014). Thus, any biological factor that significantly influences this construct may represent a potential biomarker for lifelong health. The biological mechanisms underlying the association between rs17268988 genotype and intra‐individual reaction time variability will also warrant investigation. There is some evidence that DHEA(S) levels are associated with decline in cognitive performance across aging in humans (Maggio et al., 2015) and rodents (Chen, Tseng, Wang, & Wang, 2014). At the neuroanatomical level, intra‐individual variability in reaction time has been most robustly associated with white matter volume (Nilsson, Thomas, O'Brien, & Gallagher, 2014; Walhovd & Fjell, 2007); *STS* is expressed within the white matter of the human brain, albeit at relatively low levels (Steckelbroeck et al., 2004; Stergiakouli et al., 2011). Hence, functional neuroimaging studies in man and mouse may investigate whether the lack of a functional *STS* gene, or of polymorphism at rs17268988, is associated with altered intra‐individual reaction time variability and alterations in white matter structure, and whether/how any associations are modulated by systemic DHEA(S) levels and/or age.

## Conflict of Interest

The authors declare no potential conflicts of interest.

## References

[brb3646-bib-0001] Babalola, P. A. , Fitz, N. F. , Gibbs, R. B. , Flaherty, P. T. , Li, P. K. , & Johnson, D. A. (2012). The effect of the steroid sulfatase inhibitor (p‐O‐sulfamoyl)‐tetradecanoyl tyramine (DU‐14) on learning and memory in rats with selective lesion of septal‐hippocampal cholinergic tract. Neurobiology of Learning and Memory, 98, 303–310. 23022361 10.1016/j.nlm.2012.09.003 PMC3491137

[brb3646-bib-0002] Batterham, P. J. , Bunce, D. , Mackinnon, A. J. , & Christensen, H. (2014). Intra‐individual reaction time variability and all‐cause mortality over 17 years: A community‐based cohort study. Age and Ageing, 43, 84–90. 23934546 10.1093/ageing/aft116

[brb3646-bib-0003] Brookes, K. J. , Hawi, Z. , Kirley, A. , Barry, E. , Gill, M. , & Kent, L. (2008). Association of the steroid sulfatase (STS) gene with attention deficit hyperactivity disorder. American Journal of Medical Genetics Part B: Neuropsychiatric Genetics, 147B, 1531–1535. 10.1002/ajmg.b.30873 18937300

[brb3646-bib-0004] Chatterjee, S. , Humby, T. , & Davies, W. (2016). Behavioural and psychiatric phenotypes in men and boys with X‐linked ichthyosis: Evidence from a worldwide online survey. PLoS One, 11, e0164417. 27711218 10.1371/journal.pone.0164417 PMC5053497

[brb3646-bib-0005] Chen, J. R. , Tseng, G. F. , Wang, Y. J. , & Wang, T. J. (2014). Exogenous dehydroisoandrosterone sulfate reverses the dendritic changes of the central neurons in aging male rats. Experimental Gerontology, 57, 191–202. 24929010 10.1016/j.exger.2014.06.010

[brb3646-bib-0006] Cyders, M. A. (2013). Impulsivity and the sexes: Measurement and structural invariance of the UPPS‐P Impulsive Behavior Scale. Assessment, 20, 86–97. 22096214 10.1177/1073191111428762

[brb3646-bib-0007] Davies, W. , Humby, T. , Kong, W. , Otter, T. , Burgoyne, P. S. , & Wilkinson, L. S. (2009). Converging pharmacological and genetic evidence indicates a role for steroid sulfatase in attention. Biological Psychiatry, 66, 360–367. 19251250 10.1016/j.biopsych.2009.01.001 PMC2720459

[brb3646-bib-0008] Davies, W. , Humby, T. , Trent, S. , Eddy, J. B. , Ojarikre, O. A. , & Wilkinson, L. S. (2014). Genetic and pharmacological modulation of the steroid sulfatase axis improves response control; comparison with drugs used in ADHD. Neuropsychopharmacology, 39, 2622–2632. 24842408 10.1038/npp.2014.115 PMC4140762

[brb3646-bib-0009] Dykiert, D. , Der, G. , Starr, J. M. , & Deary, I. J. (2012). Age differences in intra‐individual variability in simple and choice reaction time: Systematic review and meta‐analysis. PLoS One, 7, e45759. 23071524 10.1371/journal.pone.0045759 PMC3469552

[brb3646-bib-0010] Ho, Y. T. , Purohit, A. , Vicker, N. , Newman, S. P. , Robinson, J. J. , Leese, M. P. , … Reed, M. J. (2003). Inhibition of carbonic anhydrase II by steroidal and non‐steroidal sulphamates. Biochemical and Biophysical Research Communications, 305, 909–914. 12767917 10.1016/s0006-291x(03)00865-9

[brb3646-bib-0011] Jackson, J. D. , Balota, D. A. , Duchek, J. M. , & Head, D. (2012). White matter integrity and reaction time intraindividual variability in healthy aging and early‐stage Alzheimer disease. Neuropsychologia, 50, 357–366. 22172547 10.1016/j.neuropsychologia.2011.11.024 PMC3302689

[brb3646-bib-0012] Johnson, D. A. , Wu, T. , Li, P. , & Maher, T. J. (2000). The effect of steroid sulfatase inhibition on learning and spatial memory. Brain Research, 865, 286–290. 10821934 10.1016/s0006-8993(00)02372-6

[brb3646-bib-0013] Jonsdottir, S. , Bouma, A. , Sergeant, J. A. , & Scherder, E. J. A. (2006). Relationships between neuropsychological measures of executive function and behavioral measures of ADHD symptoms and comorbid behaviour. Archives of Clinical Neuropsychology, 21, 383–394. 16876383 10.1016/j.acn.2006.05.003

[brb3646-bib-0014] Kent, L. , Emerton, J. , Bhadravathi, V. , Weisblatt, E. , Pasco, G. , Willatt, L. R. , … Yates, J. R. (2008). X‐linked ichthyosis (steroid sulfatase deficiency) is associated with increased risk of attention deficit hyperactivity disorder, autism and social communication deficits. Journal of Medical Genetics, 45, 519–524. 18413370 10.1136/jmg.2008.057729

[brb3646-bib-0015] Lynam, D. R. , Smith, G. T. , Whiteside, S. P. , & Cyders, M. A. (2006). The UPPS‐P: Assessing five personality pathways to impulsive behavior. West Lafayette, IN: Purdue University.

[brb3646-bib-0016] Maggio, M. , De Vita, F. , Fisichella, A. , Colizzi, E. , Provenzano, S. , Lauretani, F. , … Ceda, G. P. (2015). DHEA and cognitive function in the elderly. Journal of Steroid Biochemistry and Molecular Biology, 145, 281–292. 24794824 10.1016/j.jsbmb.2014.03.014

[brb3646-bib-0017] Mueller, J. W. , Gilligan, L. C. , Idkowiak, J. , Arlt, W. , & Foster, P. A. (2015). The regulation of steroid action by sulfation and desulfation. Endocrine Reviews, 36, 526–563. 26213785 10.1210/er.2015-1036 PMC4591525

[brb3646-bib-0018] Mueller, S. T. , & Piper, B. J. (2014). The Psychology Experiment Building Language (PEBL) and PEBL test battery. Journal of Neuroscience Methods, 222, 250–259. 24269254 10.1016/j.jneumeth.2013.10.024 PMC3897935

[brb3646-bib-0019] Nicolas, L. B. , Pinoteau, W. , Papot, S. , Routier, S. , Guillaumet, G. , & Mortaud, S. (2001). Aggressive behavior induced by the steroid sulfatase inhibitor COUMATE and by DHEAS in CBA/H mice. Brain Research, 922, 216–222. 11743952 10.1016/s0006-8993(01)03171-7

[brb3646-bib-0020] Nilsson, J. , Thomas, A. J. , O'Brien, J. T. , & Gallagher, P. (2014). White matter and cognitive decline in aging: A focus on processing speed and variability. Journal of the International Neuropsychological Society, 20, 262–267. 24528516 10.1017/S1355617713001458

[brb3646-bib-0021] Pashler, H. (1984). Processing stages in overlapping tasks: Evidence for a central bottleneck. Journal of Experimental Psychology: Human Perception and Performance, 10, 358–377. 6242412 10.1037//0096-1523.10.3.358

[brb3646-bib-0022] Patton, J. H. , Stanford, M. S. , & Barratt, E. S. (1995). Factor structure of the Barratt impulsiveness scale. Journal of Clinical Psychology, 51, 768–774. 8778124 10.1002/1097-4679(199511)51:6<768::aid-jclp2270510607>3.0.co;2-1

[brb3646-bib-0023] Phillips, M. , Rogers, P. , Haworth, J. , Bayer, A. , & Tales, A. (2013). Intra‐individual reaction time variability in mild cognitive impairment and Alzheimer's disease: Gender, processing load and speed factors. PLoS One, 8, e65712. 23762413 10.1371/journal.pone.0065712 PMC3677873

[brb3646-bib-0024] Ramchurn, A. , de Fockert, J. W. , Mason, L. , Darling, S. , & Bunce, D. (2014). Intra‐individual reaction time variability affects P300 amplitude rather than latency. Frontiers in Human Neuroscience, 8, 557. 25120458 10.3389/fnhum.2014.00557 PMC4114286

[brb3646-bib-0025] Stanford, M. S. , Mathias, C. W. , Dougherty, D. M. , Lake, S. L. , Anderson, N. E. , & Patton, J. H. (2009). Fifty years of the Barratt Impulsiveness Scale: An update and review. Personality and Individual Differences, 47, 385–395.

[brb3646-bib-0026] Steckelbroeck, S. , Nassen, A. , Ugele, B. , Ludwig, M. , Watzka, M. , Reissinger, A. , … Hans, V. H. (2004). Steroid sulfatase (STS) expression in the human temporal lobe: Enzyme activity, mRNA expression and immunohistochemistry study. Journal of Neurochemistry, 89, 403–417. 15056284 10.1046/j.1471-4159.2004.02336.x

[brb3646-bib-0027] Stergiakouli, E. , Langley, K. , Williams, H. , Walters, J. , Williams, N. M. , Suren, S. , … Davies, W. (2011). Steroid sulfatase is a potential modifier of cognition in attention deficit hyperactivity disorder. Genes Brain and Behavior, 10, 334–344. 10.1111/j.1601-183X.2010.00672.x PMC3664024 21255266

[brb3646-bib-0028] Trent, S. , Dean, R. , Veit, B. , Cassano, T. , Bedse, G. , Ojarikre, O. A. , … Davies, W. (2013). Biological mechanisms associated with increased perseveration and hyperactivity in a genetic mouse model of neurodevelopmental disorder. Psychoneuroendocrinology, 38, 1370–1380. 23276394 10.1016/j.psyneuen.2012.12.002 PMC3690523

[brb3646-bib-0029] Trent, S. , Fry, J. P. , Ojarikre, O. A. , & Davies, W. (2014). Altered brain gene expression but not steroid biochemistry in a genetic mouse model of neurodevelopmental disorder. Molecular Autism, 5, 21. 24602487 10.1186/2040-2392-5-21 PMC3946266

[brb3646-bib-0030] Verbruggen, F. , Aron, A. R. , Stevens, M. A. , & Chambers, C. D. (2010). Theta burst stimulation dissociates attention and action updating in human inferior frontal cortex. Proceedings of the National Academy of Sciences of the United States of America, 107, 13966–13971. 20631303 10.1073/pnas.1001957107 PMC2922216

[brb3646-bib-0031] Verbruggen, F. , & Logan, G. D. (2009). Models of response inhibition in the stop‐signal and stop‐change paradigms. Neuroscience and Biobehavioral Reviews, 33, 647–661. 18822313 10.1016/j.neubiorev.2008.08.014 PMC2696813

[brb3646-bib-0032] Walhovd, K. B. , & Fjell, A. M. (2007). White matter volume predicts reaction time instability. Neuropsychologia, 45, 2277–2284. 17428508 10.1016/j.neuropsychologia.2007.02.022

[brb3646-bib-0033] Winterer, G. , Mittelstrass, K. , Giegling, I. , Lamina, C. , Fehr, C. , Brenner, H. , … Rujescu, D. (2010). Risk gene variants for nicotine dependence in the CHRNA5‐CHRNA3‐CHRNB4 cluster are associated with cognitive performance. American Journal of Medical Genetics Part B: Neuropsychiatric Genetics, 153B, 1448–1458. 20886544 10.1002/ajmg.b.31126

[brb3646-bib-0100] Yue, X. H. , Tong, J. Q. , Wang, Z. J. , Zhang, J. , Liu, X. , Liu, X.J. , … Qi, J. S. (2016). Steroid sulfatase inhibitor DU‐14 protects spatial memory and synaptic plasticity from disruption by amyloid beta protein in male rats. Hormones and Behavior, 83, 83–92. 27222435 10.1016/j.yhbeh.2016.05.019

